# Trends in Repeat Births and Use of Postpartum Contraception Among Teens — United States, 2004–2015

**DOI:** 10.15585/mmwr.mm6616a3

**Published:** 2017-04-28

**Authors:** Deborah L. Dee, Karen Pazol, Shanna Cox, Ruben A. Smith, Katherine Bower, Martha Kapaya, Amy Fasula, Ayanna Harrison, Charlan D. Kroelinger, Denise D’Angelo, Leslie Harrison, Emilia H. Koumans, Nikki Mayes, Wanda D. Barfield, Lee Warner

**Affiliations:** ^1^Division of Reproductive Health, National Center for Chronic Disease Prevention and Health Promotion, CDC; ^2^Oak Ridge Institute for Science and Education.

Teen* childbearing (one or more live births before age 20 years) can have negative health, social, and economic consequences for mothers and their children (*1*). Repeat teen births (two or more live births before age 20 years) can constrain the mother’s ability to take advantage of educational and workforce opportunities (*2*), and are more likely to be preterm or of low birthweight than first teen births (*3*). Despite the historic decline in the U.S. teen birth rate during 1991–2015, from 61.8 to 22.3 births per 1,000 females aged 15–19 years (*4*), many teens continue to have repeat births (*3*). The American College of Obstetricians and Gynecologists and the American Academy of Pediatrics both recommend that clinicians counsel women (including teens) during prenatal care about birth spacing and postpartum contraceptive use (*5*), including the safety and effectiveness of long-acting reversible methods that can be initiated immediately postpartum. To expand upon prior research assessing patterns and trends in repeat childbearing and postpartum contraceptive use among teens with a recent live birth (i.e., 2–6 months after delivery) (*3*), CDC analyzed data from the National Vital Statistics System natality files (2004 and 2015) and the Pregnancy Risk Assessment Monitoring System (PRAMS; 2004–2013). The number and proportion of teen births that were repeat births decreased from 2004 (82,997; 20.1%) to 2015 (38,324; 16.7%); in 2015, the percentage of teen births that were repeat births varied by state from 10.6% to 21.4%. Among sexually active teens with a recent live birth, postpartum use of the most effective contraceptive methods (intrauterine devices and contraceptive implants) increased from 5.3% in 2004 to 25.3% in 2013; however, in 2013, approximately one in three reported using either a least effective method (15.7%) or no method (17.2%). Strategies that comprehensively address the social and health care needs of teen parents can facilitate access to and use of effective methods of contraception and help prevent repeat teen births.

National Vital Statistics System natality files, compiled annually by CDC’s National Center for Health Statistics, include demographic information such as maternal age, race, and Hispanic ethnicity for all births in the 50 states and the District of Columbia.^†^ CDC analyzed national and state-specific natality data for 2004 and 2015 for teens aged 15–19 years in which information about the number of previous live births was available. The total number of births with known birth order to teens aged 15–19 years was 413,144 in 2004 and 228,862 in 2015, representing ≥99% of births in this age group for these years. The percentage change from 2004 to 2015 in teen births that were repeat teen births, overall and for each state, was evaluated using a two-sided Z-test, with significance set at p<0.05.

PRAMS is an ongoing population-based surveillance system designed to monitor selected self-reported behaviors and experiences before, during, and after pregnancy among women with a recent live birth (*6*). To measure postpartum contraceptive use among teens aged <20 years,^§^ CDC analyzed PRAMS data from 30 states^¶^ and New York City (states) that met survey response rate criteria of 60%** in 2013, and 5 states^††^ that met response rate thresholds continuously during 2004–2013. Contraceptive methods were placed in three tiers of effectiveness based on the percentage of users who experience pregnancy during the first year of typical use: most effective (<1%),^§§^ moderately effective (6%–10%),^¶¶^ and least effective (>10%)*** (*7*). Teens reporting multiple contraceptive methods were categorized by the most effective method used. Trends in postpartum contraceptive use were analyzed in 2-year increments to account for the complex sampling design of PRAMS. CDC calculated weighted prevalence estimates and 95% confidence intervals and used chi-squared analyses to measure differences in postpartum contraceptive use, and tested for linear and quadratic changes in contraceptive use over time.

## Repeat Teen Births: 2015 and Change from 2004 to 2015

In 2015, among 413,144 births to teens aged 15–19 years, 38,324 (16.7%) were repeat births (Supplementary Table 1; https://stacks.cdc.gov/view/cdc/). The prevalence of teen births that were repeat births was highest among Hispanics (18.7%), followed by non-Hispanic black (black) (17.9%), and non-Hispanic white (white) (14.3%) births. The proportion of teen births that were repeat births varied by state, from 10.6% in Vermont to 21.4% in the District of Columbia.

**FIGURE 1:**
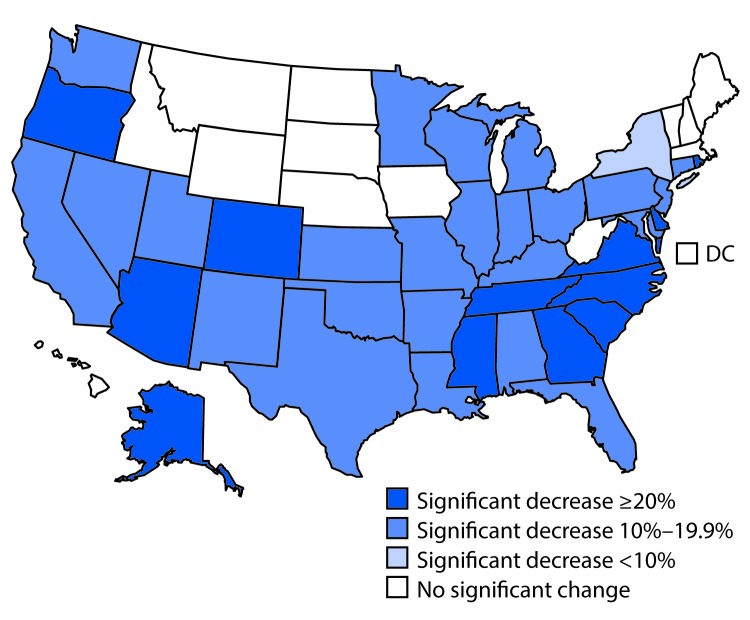
Percent change in repeat teen births* —United States, 2004–2015 * Repeat teen births are two or more live births to a mother aged <20 years ^†^ Data for 2004 and 2015 downloaded from CDC WONDER (https://wonder.cdc.gov).

Overall, the number of repeat teen births declined 53.8%, from 82,997 in 2004 to 38,324 in 2015. In addition, the percentage of teen births that were repeat births decreased 16.9%, from 20.1% in 2004 to 16.7% in 2015. By race/ethnicity, the largest declines in the percentage of teen births that were repeat births occurred among blacks (21.8%), followed by Hispanics (16.8%), and whites (13.9%). By age, declines in the percentage of teen births that were repeat births occurred both among teens aged 15–17 years (23.8%) and 18–19 years (19.7%). From 2004 to 2015, 35 states experienced a significant decline in the percentage of teen births that were repeat births; of the 35 states, 12 experienced declines of >20%, and none experienced a significant increase (Figure 1).

## Current Postpartum Contraceptive Use — 31 States, 2013

In 2013, among teens with a recent live birth, 82.8% reported postpartum using contraception, with 26.9% using most effective, 40.2% using moderately effective, and 15.7% using least effective methods (Table 1). By state, the percentage of teens with a recent live birth who reported using a most effective method postpartum ranged from 11.4% in New York City to 51.5% in Colorado, and the percentage using no method ranged from 4.9% in Vermont to 33.8% in New Jersey.

## Trends in Postpartum Contraceptive Use Among Teens in Five States During 2004–2013

**FIGURE 2:**
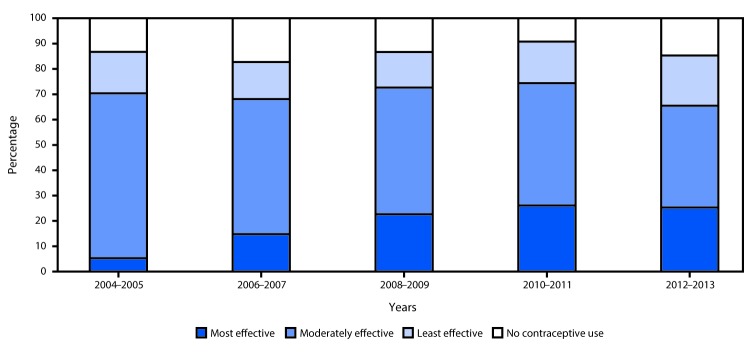
Trends and distribution of postpartum contraception method use* among teens^†^ — Pregnancy Risk Assessment Monitoring System, five states,^§^ 2004–2013 * Methods categorized by effectiveness, as determined by the percentage of females who experience pregnancy during the first year of typical use as the following: *most effective* (contraceptive implant and intrauterine device) (<1%); *moderately effective* (oral contraceptive pill, an injectable [e.g., Depo-Provera], birth control patch, and vaginal ring) (6–10%); and *least effective* (condom, diaphragm, cervical cap, contraceptive sponge, rhythm method/natural family planning, the “morning after pill,” withdrawal, and “other” responses that could not be categorized to a more effective category) (>10%); also includes measure of teen mothers who report no postpartum contraceptive use. ^†^ For this report, the term “teens” refers to persons aged <20 years.

Among the five states that continuously collected data on teens’ use of postpartum contraception, the use of any method remained relatively stable during 2004–2013 (range by 2–year increment = 82.7% to 90.8%), but the distribution of contraceptive methods used changed over time (Figure 2) (Supplementary Table 2; https://stacks.cdc.gov/view/cdc/45185). From 2004–2005 to 2012–2013, use of the most effective reversible methods increased significantly, from 5.3% to 25.3%, and use of moderately effective methods decreased significantly, from 65.1% to 40.2%; use of least effective methods and no method did not change significantly.

## Discussion

From 2004 to 2015, the number and proportion of teen births in the United States that were repeat births decreased 53.8% and 16.9%, respectively. Further, the percentage of teens with a recent live birth who used a most effective contraceptive method postpartum increased substantially during 2004-2013, from 5.3% to 25.3%. This increase in teens’ use of the most effective contraceptive methods mirrors the pattern observed among all reproductive-aged women who participated in the National Survey of Family Growth during this period (*8*). Despite these improvements, in 2015, one in six teen births was a repeat birth, and in 2013, one in three teens with a recent live birth used either a least effective method or no method of contraception.

These results demonstrate a shift in the distribution of the types of reversible contraception used by teens with a recent live birth; use of the most effective contraceptive methods increased, with a concomitant decline in use of moderately effective methods, and no significant change in use of least effective methods or no method. Recently developed clinical performance measures for contraceptive care have established the use of most or moderately effective methods as an indicator of quality family planning service provision and can help identify populations where a need exists for improving access to contraception in the postpartum period.^†††^ Strategies for increasing access to postpartum contraception among parenting teens include provision of youth-friendly services that address adolescent confidentiality concerns, adequate client-centered counseling, and increased provider and consumer awareness of the full range of contraceptive methods (*9*).

Previous analyses have found wide variation in postpartum contraceptive use among teens across states (*3*,*10*). Although states vary in sociodemographic factors that might influence repeat births among teens, variation also exists in the implementation of measures designed to increase access to and use of immediate postpartum long-acting reversible contraception among women, including teens (*11*). For example, some states have implemented policies that provide enhanced reimbursement of immediate postpartum long-acting reversible contraception insertion for Medicaid-enrolled mothers, thereby removing health care system barriers.^§§§^^,^^¶¶¶^^,^**** In addition, some states provide support services to teen parents, such as home visiting programs,^††††^^,^^§§§§^ which have been found to reduce repeat teen births.

The findings in this report are subject to at least five limitations. First, although contraceptive effectiveness is dependent on both consistent and correct use, particularly for the least effective methods, neither of these attributes was measured through PRAMS questions. Second, data on postpartum contraceptive use were only available in the PRAMS states with response rates that met the reporting threshold; therefore, findings might not be generalizable to all states. Third, because of small sample sizes, state-level prevalence estimates for certain categories of contraceptive effectiveness were unstable, with wide and overlapping confidence intervals. Fourth, PRAMS data are self-reported and thus potentially subject to social desirability bias. Finally, although the rate of repeat teen births (per 1,000 female teens) might better reflect changes in the population of females at risk for having a repeat teen birth, this report highlights strategies to reduce the proportion of teen births that are repeat births.

This report found continued decreases in repeat teen births and increases in use of the most effective contraceptive methods among teens with a recent live birth. At the same time, use of moderately effective methods declined and use of least effective methods or no method remained stable. Further reducing repeat births among teens requires ensuring access to the full range of Food and Drug Administration–approved methods of contraception during the postpartum period (*11*) and increased use of moderately effective and most effective methods.

**Summary**

**What is known about this topic?**

Despite record declines in the rate of births among teens, many women continue to have repeat births during their teen years. Use of postpartum contraception can help teens avoid repeat births.

**What is added by this report?**

From 2004 to 2015, the number and percentage of teen births that were repeat births decreased 53.8% and 16.9%, respectively; in 2015, the percentage of teen births that were repeat births varied by state from 10.6% to 21.4%. Among teens with a recent live birth, use of the most effective contraceptive methods postpartum increased substantially, from 5.3% in 2004 to 25.3% in 2013; however, in 2013, approximately one in three teens with a recent live birth reported using a least effective contraceptive method or no method postpartum.

**What are the implications for public health practice?**

Strategies that comprehensively address the social and health care needs of parenting teens, such as provision of youth-friendly services, adequate client-centered counseling, and promotion of provider and consumer awareness of the range of contraceptive methods, can help improve use of effective contraception postpartum and prevent repeat teen births.

**TABLE:** Postpartum contraceptive use among teens,* by selected characteristics — Pregnancy Risk Assessment Monitoring System, 31 states,^†^ 2013

Characteristic (No.)	Overall		Postpartum contraceptive use								Chi-squared p-value
			Most effective^§^		Moderately effective^§^		Least effective^§^		No contraceptive use^§^		
	No.	% (95% CI)^¶^	No.	% (95% CI)^¶^	No.	% (95% CI)^¶^	No.	% (95% CI)^¶^	No	% (95% CI) ^¶^	
**Total (2,518)**	—	100 (–)	652	26.9 (23.8–30.2)	984	40.2 (36.7–43.9)	415	15.7 (13.3–18.5)	467	17.2 (14.7–20.0)	—
**Maternal age (yrs) (2,518)**											0.5964
≤17	744	29.0 (25.8–32.5)	204	25.4 (20.0–31.6)	309	43.9 (37.2–50.8)	101	13.8 (9.7–19.3)	130	16.9 (12.4–22.6)	
18–19	1,774	71.0 (67.5–74.2)	448	27.5 (23.8–31.5)	675	38.7 (34.6–43.1)	314	16.5 (13.6–19.8)	337	17.3 (14.4–20.6)	
**Previous live birth (2,499)**											0.8867
No	2,135	86.1 (83.5–88.4)	546	26.5 (23.2–30.2)	849	40.7 (36.8–44.6)	343	15.7 (13.1–18.8)	397	17.1 (14.4–20.2)	
Yes	364	13.9 (11.6–16.5)	102	29.5 (21.8–38.6)	126	36.9 (28.2–46.6)	67	15.6 (10.2–23.1)	69	17.9 (12.1–25.8)	
**Race/Ethnicity (2,455)**											0.0003
White, non-Hispanic	839	45.9 (42.2–49.6)	193	26.9 (22.1–32.3)	362	40.2 (34.8–45.9)	154	18.5 (14.6–23.1)	130	14.4 (11.0–18.7)	
Black, non-Hispanic	598	21.9 (19.0–25.2)	115	15.1 (10.2–21.6)	281	50.1 (42.0–58.2)	88	12.6 (8.1–18.9)	114	22.3 (16.0–30.2)	
Hispanic	656	23.6 (20.9–26.7)	223	36.1 (29.9–42.7)	206	34.6 (28.1–41.7)	101	13.0 (9.4–17.8)	126	16.3 (12.2–21.5)	
**Marital status (2,518)**											0.0627
Married	276	10.9 (9.0–13.2)	69	19.7 (14.0–27.0)	86	35.8 (26.2–46.6)	67	21.8 (15.0–30.6)	54	22.7 (15.1–32.5)	
Other	2,242	89.1 (86.8–91.0)	583	27.8 (24.4–31.4)	898	40.8 (37.0–44.7)	348	15.0 (12.4–17.9)	413	16.5 (13.9–19.5)	
**State^†^ (2,518)**											<0.0001
Alaska	92	1.0 (0.7–1.2)	31	32.6 (22.1–45.2)	18	22.7 (13.6–35.5)	20	21.9 (13.2–34.2)	23	22.8 (13.9–35.0)	
Arkansas	98	2.4 (1.7–3.5)	9	13.7 (4.4–35.1)	52	53.1 (34.4–71.0)	17	17.8 (7.1–38.0)	20	15.4 (6.6–32.0)	
Colorado	90	3.8 (2.8–5.2)	37	51.5 (36.1–66.6)	24	29.7 (17.4–45.9)	15	11.1 (5.4–21.4)	14	7.7 (3.5–16.2)	
Delaware	49	0.6 (0.5–0.8)	7	15.3 (7.4–29.0)	30	63.5 (48.9–76.1)	4	6.1 (2.1–16.2)	8	15.1 (7.5–28.0)	
Georgia	50	5.4 (3.6–8.1)	13	39.9 (21.0–62.3)	23	31.9 (15.7–54.2)	7	13.4 (3.9–36.7)	7	14.8 (4.8–37.4)	
Hawaii	73	1.0 (0.7–1.4)	13	15.8 (7.4–30.6)	30	36.6 (23.4–52.3)	14	18.2 (9.2–32.7)	16	29.4 (16.6–46.5)	
Illinois	71	11.3 (8.8–14.3)	16	19.7 (10.8–33.0)	33	52.7 (39.3–65.8)	13	12.6 (6.6–23.0)	9	15.0 (7.5–27.5)	
Iowa	95	2.3 (1.6–3.3)	27	13.7 (6.4–26.8)	32	47.0 (29.6–65.2)	19	20.0 (9.0–38.7)	17	19.3 (8.5–38.2)	
Maine	47	0.7 (0.5–0.9)	14	32.6 (18.5–50.6)	18	34.3 (20.0–52.1)	7	13.1 (5.1–29.5)	8	20.1 (9.4–37.9)	
Maryland	35	2.3 (1.5–3.4)	4	15.8 (5.4–38.0)	14	35.0 (18.5–56.0)	8	21.7 (9.2–42.9)	9	27.6 (12.9–49.5)	
Massachusetts	54	2.6 (1.8–3.7)	22	36.4 (21.9–54.0)	18	40.2 (23.7–59.4)	5	6.1 (2.4–14.6)	9	17.2 (7.0–36.4)	
Michigan	141	7.7 (5.9–10.1)	31	18.6 (10.0–31.9)	73	55.8 (41.4–69.3)	23	19.2 (9.9–34.1)	14	6.4 (3.5–11.4)	
Minnesota	46	2.6 (1.8–3.8)	18	28.9 (15.9–46.8)	13	30.2 (15.9–49.8)	6	17.4 (6.1–40.7)	9	23.4 (10.3–44.8)	
Missouri	88	6.5 (5.2–8.2)	33	27.2 (18.2–38.6)	24	27.7 (18.1–39.9)	17	24.7 (15.5–36.9)	14	20.4 (12.1–32.2)	
Nebraska	85	1.4 (1.1–1.9)	23	30.9 (20.0–44.4)	27	30.9 (20.3–44.0)	14	17.6 (9.5–30.3)	21	20.7 (12.4–32.4)	
New Hampshire	36	0.9 (0.6–1.3)	13	43.0 (24.2–64.0)	13	27.7 (13.7–48.0)	6	17.6 (7.0–37.5)	4	11.7 (3.2–35.2)	
New Jersey	32	2.0 (1.3–2.9)	5	20.3 (7.8–43.6)	9	25.3 (12.0–45.6)	8	20.7 (9.0–40.7)	10	33.8 (16.8–56.2)	
New Mexico	148	3.0 (2.5–3.6)	50	39.3 (30.8–48.5)	54	30.5 (23.3–38.7)	18	12.3 (7.4–19.6)	26	18.0 (12.0–26.0)	
New York	326	5.4 (4.0–7.4)	58	13.6 (6.3–26.7)	133	49.4 (33.8–65.2)	63	8.9 (6.0–13.1)	72	28.1 (15.8–45.0)	
New York City	54	3.5 (2.4–5.1)	7	11.4 (3.8–29.3)	28	57.0 (38.4–73.8)	7	17.6 (7.3–36.8)	12	14.0 (5.7–30.6)	
Oklahoma	108	4.1 (2.9–5.6)	21	32.2 (18.4–50.0)	46	39.1 (24.2–56.3)	15	14.7 (6.4–30.5)	26	14.0 (6.6–27.0)	
Oregon	77	2.7 (2.0–3.7)	35	46.9 (31.7–62.7)	22	27.8 (15.4–44.7)	7	10.5 (3.7–26.0)	13	14.9(7.3–27.9)	
Pennsylvania	43	6.4 (4.6–8.9)	9	23.2 (11.6–41.2)	20	41.9 (26.1–59.6)	8	17.5 (7.9–34.3)	6	17.4 (7.2–36.3)	
Rhode Island	50	0.5 (0.4–0.8)	19	35.9 (22.2–52.3)	20	38.9 (24.6–55.4)	3	7.8 (2.6–21.0)	8	17.4 (8.0–33.9)	
Tennessee	56	7.6 (5.6–10.3)	12	29.9 (16.8–47.5)	29	42.1 (26.9–59.0)	8	17.0 (7.6–33.8)	7	10.9 (4.0–26.4)	
Utah	74	1.8 (1.4–2.3)	28	35.7 (25.0–48.0)	28	34.7 (24.1–47.0)	5	6.3 (2.6–14.7)	13	23.4 (12.7–39.1)	
Vermont	43	0.3 (0.2–0.4)	17	40.3 (25.4–57.2)	15	38.0 (23.4–55.1)	8	16.9 (7.8–32.7)	3	4.9 (1.2–18.5)	
Washington	41	4.0 (2.7–5.8)	13	37.8 (21.1–57.8)	11	26.1 (12.4–46.7)	7	12.8 (4.3–32.6)	10	23.4 (10.9–43.1)	
West Virginia	153	2.1 (1.7–2.6)	19	11.9 (6.3–21.1)	77	50.6 (39.5–61.6)	30	18.9 (11.6–29.3)	27	18.6 (11.4–29.0)	
Wisconsin	129	3.6 (2.7–4.9)	41	32.9 (20.3–48.5)	38	24.7 (14.5–38.8)	25	19.0 (9.9–33.5)	25	23.4 (13.2–37.9)	
Wyoming	34	0.5 (0.4–0.8)	7	21.2 (9.0–42.4)	12	32.9 (16.7–54.4)	8	28.2 (13.2–50.4)	7	17.6 (6.7–38.8)	

**Abbreviation:** CI = confidence interval.

* For this report, the term “teens” refers to persons aged <20 years.

**^†^** “States” refer to 30 states (Alaska, Arkansas, Colorado, Delaware, Georgia, Hawaii, Illinois, Iowa, Maine, Maryland, Massachusetts, Michigan, Minnesota, Mississippi, Nebraska, New Hampshire, New Jersey, New Mexico, New York, Oklahoma, Oregon, Pennsylvania, Rhode Island, Tennessee, Utah, Vermont, Washington, West Virginia, Wisconsin, and Wyoming) and New York City.

^§^ Methods categorized by effectiveness, as determined by the percentage of females who experience pregnancy during the first year of typical use: *most effective* (contraceptive implant and intrauterine device) (<1%); *moderately effective* (oral contraceptive pill, an injectable (e.g., Depo–Provera), birth control patch, and vaginal ring) (6%–10%); and *least effective* (condom, diaphragm, cervical cap, contraceptive sponge, rhythm method/natural family planning, the “morning after pill,” withdrawal, and “other” responses that could not be categorized to a more effective category) (>10%); also includes measure of teen mothers who report no postpartum contraceptive use.

^¶^ Weighted percent.

## Footnotes

* For this report, the term “teen” refers to a person aged <20 years.

† CDC National Center for Health Statistics, Division of Vital Statistics, Natality public-use data.

§ To measure postpartum contraceptive respondents were asked, “Are you or your husband or partner doing anything now to keep from getting pregnant?” and if the respondent answered affirmatively, she was then asked, “What kind of birth control are you or your husband or partner using now to keep from getting pregnant?” Analyses excluded teens who were currently pregnant, not sexually active, or used nonreversible methods of contraception (male or female sterilization).

¶ The thirty states (Alaska, Arkansas, Colorado, Delaware, Georgia, Hawaii, Illinois, Iowa, Maine, Maryland, Massachusetts, Michigan, Minnesota, Mississippi, Nebraska, New Hampshire, New Jersey, New Mexico, New York, Oklahoma, Oregon, Pennsylvania, Rhode Island, Tennessee, Utah, Vermont, Washington, West Virginia, Wisconsin, and Wyoming) and New York City are hereafter referred to as “states.”

** PRAMS response rates required for states to be included in analyses were 70% for 2004–2006, 65% for 2007–2011, and 60% for 2012–2013.

†† Arkansas, Michigan, Nebraska, Oregon, and Rhode Island.

§§ Contraceptive implants and intrauterine devices.

¶¶ Oral contraceptive pills, injectables (e.g., Depo-Provera), birth control patches, and vaginal rings.

*** Condoms, diaphragms, cervical caps, contraceptive sponges, rhythm method/natural family planning, the “morning after pill,” withdrawal, and “other” responses that could not be categorized into a more effective category.

††† https://www.hhs.gov/opa/performance-measures/index.html.

§§§ Additional information about state Medicaid approaches to improve access to LARC is available at https://www.medicaid.gov/federal-policy-guidance/downloads/cib040816.pdf.

¶¶¶ Additional information about Medicaid reimbursement of postpartum LARC in the hospital setting is available at http://www.acog.org/About-ACOG/ACOG-Departments/Long-Acting-Reversible-Contraception/Immediate-Postpartum-LARC-Medicaid-Reimbursement.

**** Additional information about the Association of State and Territorial Health Officials’ state learning communities and state initiatives to improve access to contraception is available at http://www.astho.org/Programs/Maternal-and-Child-Health/Long-Acting-Reversible-Contraception-LARC/.

†††† Additional information about the federal home visiting program is available at https://mchb.hrsa.gov/maternal-child-health-initiatives/home-visiting-overview.

§§§§ Additional information about programs for expectant and parenting teens is available at https://www.hhs.gov/ash/oah/grant-programs/pregnancy-assistance-fund/index.html.
