# *Erratum*: Vol. 66, No. 16

**DOI:** 10.15585/mmwr.mm6646a6

**Published:** 2017-11-24

In the report “Trends in Repeat Births and Use of Postpartum Contraception Among Teens — United States, 2004–2015,” on page 422, the last sentence of the second column should have read “Trends in postpartum contraceptive use were analyzed in 2-year increments **using statistical software** to account for the complex sampling design of PRAMS.” In addition, the fourth footnote on page 422 should have read “^¶ ^The thirty states (Alaska, Arkansas, Colorado, Delaware, Georgia, Hawaii, Illinois, Iowa, Maine, Maryland, Massachusetts, Michigan, Minnesota, **Missouri**, Nebraska, New Hampshire, New Jersey, New Mexico, New York, Oklahoma, Oregon, Pennsylvania, Rhode Island, Tennessee, Utah, Vermont, Washington, West Virginia, Wisconsin, and Wyoming) and New York City are hereafter referred to as “states.””

On page 423, the first sentence of the first paragraph under “Repeat Teen Births: 2015 and Change from 2004 to 2015,” should have read “In 2015, among **228,862** births to teens aged 15–19 years, 38,324 (16.7%) were repeat births (Supplementary Table 1; https://stacks.cdc.gov/view/cdc/45184).”

On page 424, the second footnote of the Table should have read “^† ^“States” refer to 30 states (Alaska, Arkansas, Colorado, Delaware, Georgia, Hawaii, Illinois, Iowa, Maine, Maryland, Massachusetts, Michigan, Minnesota, **Missouri**, Nebraska, New Hampshire, New Jersey, New Mexico, New York, Oklahoma, Oregon, Pennsylvania, Rhode Island, Tennessee, Utah, Vermont, Washington, West Virginia, Wisconsin, and Wyoming) and New York City.”

